# A Practical Approach for Determination of Thermal Stress and Temperature-Dependent Material Properties in Multilayered Thin Films

**DOI:** 10.1021/acsami.4c03166

**Published:** 2024-06-10

**Authors:** Yanqiao Yang, Andreas Winkler, Atefeh Karimzadeh

**Affiliations:** Leibniz IFW Dresden, SAWLab Saxony, Institute for Emerging Electronic Technologies (IET), Group “Acoustic Microsystems”, Helmholtzstr. 20, 01069 Dresden, Germany

**Keywords:** thermo-mechanical properties, multilayer-adapted Stoney equation, curvature measurement method, thermal stress formula, indirect method, temperature-dependent Young’s modulus

## Abstract

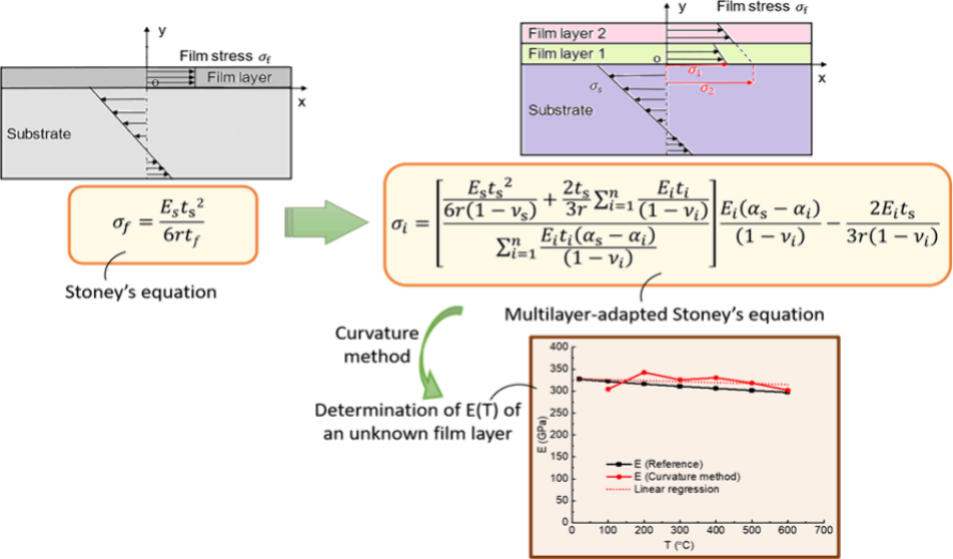

Multilayered thin films are essential to most microelectro-mechanical systems (MEMSs). The reliability and predictability of the behavior of such systems, especially when intended for usage at high temperatures or in harsh environments, demand the consideration of thermo-mechanical properties of the individual films of the multilayer arrangement during the design stage. This paper introduces a newly derived analytical model for the convenient indirect determination of the temperature-dependent Young’s modulus and the thermally induced stress of individual layers within a multilayered thin film system, i.e., a multilayer-adapted Stoney equation. It is based on sample curvature measurement and requires data from only a single experiment. Experimental and numerical investigations of the new models are carried out using a five-layered sample of a RuAl metallization system developed for wireless high-temperature acoustic sensing. The results highlight the usability of the new model in practical MEMS analysis, enabling insights into complex layer stacks by overcoming current experimental limitations.

## 1 Introduction

Multilayered thin film systems consisting of a number of thin film layers deposited on a substrate are widely implemented in microelectro-mechanical systems (MEMSs) including surface acoustic wave (SAW) devices for high-temperature (ex. temperature sensors) or low temperature (ex. deicing sensors and actuators) implementations.^1,2^ Since the material properties of films and the substrate in multilayered thin film systems are generally different, residual thermal stress will be generated during temperature changes, which leads to the dysfunction or failure of SAW devices (e.g., signal distortion in SAW sensors or damages such as delamination of film layers).^3−7^ Furthermore, the properties of thin films depend on the method and conditions of their deposition on a substrate. The changing film system properties can lead to unpredictable behavior of MEMSs. Therefore, the design of multilayered thin film systems in variable temperature environments needs high orders of reliability, which requires knowing the thermo-mechanical properties of thin film layers, especially the temperature-dependent Young’s modulus, as well as thermal stresses in each layer of the films.

For the film stress analysis, Stoney^8^ has derived an equation to calculate the residual film stress in a single film layer on a substrate. Although the uniaxial stress assumption in his model may not be applicable in practical situations since the film has two dimensions and is in a plane stress state, the Stoney equation remains a useful tool for estimating stress in thin films on a substrate.^9^ After considering the biaxial stress state in the model, other researchers modified Stoney’s equation, making it closer to reality.^9−12^ For multilayered thin films, the stress equation is obtained by Hsueh et.al.,^13,14^ where a closed-form solution and a simplified solution are provided. However, the closed-form solution is too complicated for engineering calculations, and the simplified solution requires multiple experiments to obtain the required parameters. Alternatively, the modified Stoney equation for the biaxial stress state can be applied directly to multilayered thin films as a whole stack to acquire the average thermal stress throughout the film layers.^9,15^ This approach is engineering-doable without multiple experiments, but the detailed distribution of thermal stress within film layers remains unknown.

On the other hand, determining temperature-dependent properties such as Young’s modulus of thin films can be challenging as no standard method exists. Conventional tensile test machines fail to obtain Young’s modulus of films with a thickness of less than 100 nm. Although the development of nanoindentation instruments makes it possible to obtain Young’s modulus of thin films at temperatures up to 1100 °C, there are still some limitations such as the requirement of a very smooth surface and the accessibility of the indentation area, thermal drifts, etc.^16,17^ Nanoindentation instruments are still not suitable for thin film materials with such a low thickness (e.g., 20 nm) considering the unknown tip shape on the nanometer range and the requirement to limit penetration to 10% of the film thickness (i.e., 2 nm for the film with a thickness of 20 nm) to exclude the substrate influence on the measured values. Additionally, determining Young’s modulus of each layer in a multilayer thin film system using the nanoindentation method would be extremely time-consuming and very complicated, which requires special calculations out of the scope of this research.

This work focuses on acquiring the thermal stress within each layer of films in multilayered thin film systems during temperature changes. In order to avoid the drawbacks of conventional methods, a new and simple analytical thermal stress equation for multilayered thin film systems is derived, which we refer to as the “multilayer-adapted Stoney equation”. To calculate the thermal stress using this equation, only a single curvature experiment of the sample prepared from the whole stack is required. A five-layered sample of RuAl metallization system^18−20^ was sputter-deposited as a multilayered thin film system and analyzed. To acquire the thermal stress or mechanical properties of thin films, the curvature variations of the sample under thermal load are determined by an in situ multibeam optical sensor (MOS) system in an ultrahigh vacuum (UHV) pressure chamber equipped with an automated heating system.^15^ In addition, a numerical model of the multilayered thin films system is built and validated by the experimental curvature results in a temperature range from 20 to 600 °C. The stress in each film layer obtained from the validated numerical simulation is compared with the results calculated from the multilayer-adapted Stoney equation.

Based on the multilayer-adapted Stoney equation, an indirect method for temperature-dependent Young’s modulus acquisition is developed and validated by a reference material with known temperature-dependent Young’s modulus. This technique introduces a new way of determining Young’s modulus of thin films at different temperatures (especially at high temperatures) by measuring the sample curvature, where traditional methods like the tensile test or nanoindentation are not suitable.

## 2 Stress State Analysis and Model Derivation

The derivation of the multilayer-adapted Stoney equation as a new and simple method for the calculation of the residual thermal stress in each layer of the multilayered thin film system is explained in this chapter.

**Figure 1:**
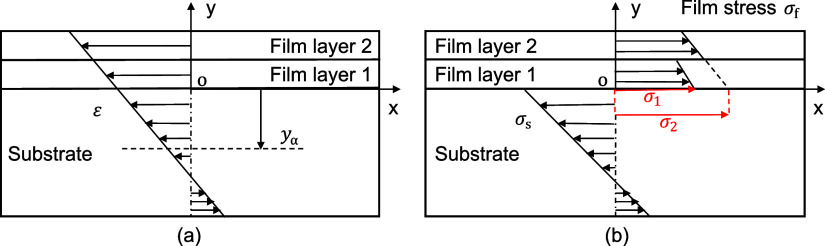
Strain and stress distribution of a left-half sample of multilayered thin film system under thermal load: (a) strain state analysis; (b) stress state analysis.

Considering Euler–Bernoulli beam assumption and displacement continuity, the strain (ε) at the left-side cross-section of the middle of the multilayered thin film system (at location *y*) shown in Figure 1a can be separated into an extension part and a bending part as
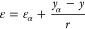
1where ε_α_ is a general extension strain, *y*_α_ is a general bending axis location, and *r* is the radius of curvature. It is important to mention that ε_α_ does not have to be equal to the thermal expansion strain and *y*_α_ does not have to be the same as the neutral surface location. They are all general cases.

To express the total strain (ε) explicitly, the constitutive equation
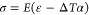
2is considered and the moment equilibrium is applied on the origin of *XY* axis (i.e., point o in Figure 1b) as
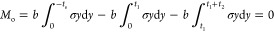
3
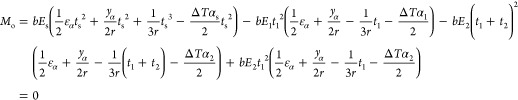
4

In most engineering implementations, including MEMS, the film thickness *t*_*i*_ (*i* from 1 to n) is much smaller than the substrate thickness *t*_s_. In this study, the thickness of the substrate is more than 1000 times larger than the film layer thickness. Therefore, in eq 4, the terms including the film thickness are negligible compared to the terms with substrate thickness. By applying first-order approximation, which ignores terms with  and higher order, film moment components in eq 4 can be eliminated with an error . Thus, eq 4 is simplified to eq 5.
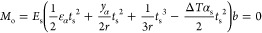
5
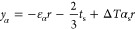
6

In eq 5, the terms *r* and ε_α_ reflect the effect of film layers on the curvature variations and the extension of the sample. By rearranging eq 5 and writing it for *y*_α_, eq 6 is obtained, which gives the relation between the general extension strain (ε_α_) and the general bending axis *y*_α_. It can be noticed that eq 6 is independent of film layers number. Inserting eq 6 into eq 1, the total strain throughout the whole system shown in Figure 1a is expressed as
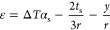
7

To simplify the following derivation, a constant representative of film stress in a thin film layer is defined. The film stress in Figure 1b can be separated into a linear part and a constant part:
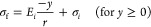
8where σ_*i*_ stands for the film stress, σ_f_, in layer *i* extended to the X axis, as shown in Figure 1b. Therefore, we refer to σ_*i*_ as the representative film stress of layer *i*. For implementations, where the stress distribution within each film layer is not of interest, the representative film stress σ_*i*_ can also be used approximately as film stress, σ_f_, with the error 

From the constitutive equation (eq 2), the total strain can also be expressed by thermal expansion strain (Δ*T*α) and the stress-induced strain () as
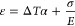
9

Inserting eq 8 into eq 9, the total strain for film layers is shown as
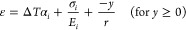
10

Combining eq 7 and eq 10, the representative film stress σ_*i*_ can be expressed as
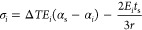
11

Inserting eq 7 into the constitutive eq (eq 2), the stress in the substrate can be expressed as
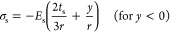
12

In order to acquire a thermal stress formula for the multilayered system expressed by sample curvature only, the temperature change (Δ*T*) in eq 11 needs to be replaced by an expression of sample curvature 1/*r*. Considering eq 8 and eq 12, for a bilayer system, the equilibrium of forces is shown as
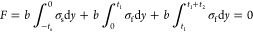
13
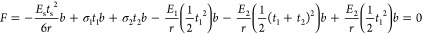
14

Generalizing eq 14 to the *n* layers case and applying first-order approximation by ignoring terms with  and higher order, eq 14 becomes
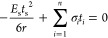
15

Inserting eq 11 into eq 15, the thermal change can be expressed by sample curvature as
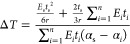
16

Thus, by inserting eq 16 into eq 11, the representative film stress σ_*i*_ can be expressed by sample curvature as
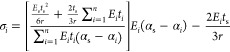
17

Inserting eq 17 into eq 8 and considering eq 12, the thermal stress distribution in the multilayered thin films system is shown as
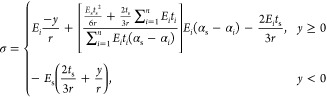
18With eq 18, the thermal stress in an *n*-layered thin film system can be acquired by the sample curvature radius *r* from a single experiment. Equation 18 is first-order accurate with an error . For engineering implementations, however, constant representatives of film stress in each layer of a multilayered system are usually of main importance. Thus, eq 17 is preferred in practice. For isotropic films, eq 17 becomes
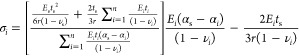
19

Comparing eq 19 to the below equation suggested by Hsueh^13^ modified by describing it in the same coordinate system as well as in isotropic condition:
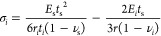
20which needs *n* curvature experiments to calculate *r*_*i*_ for each film layer in the single-layered case and an additional experiment to calculate *r* for the *n*-layered thin film system, the multilayer-adapted Stoney equation reduces the experiment number from *n* + 1 to 1. This advantage of eq 19 and 18 not only saves experimental resources and time but also provides the opportunity for in situ real-time analysis of the sample without multiple experiments, which is important for engineering applications.

## 3 Materials and Methods

### 3.1 Sample Preparation

**Figure 2:**
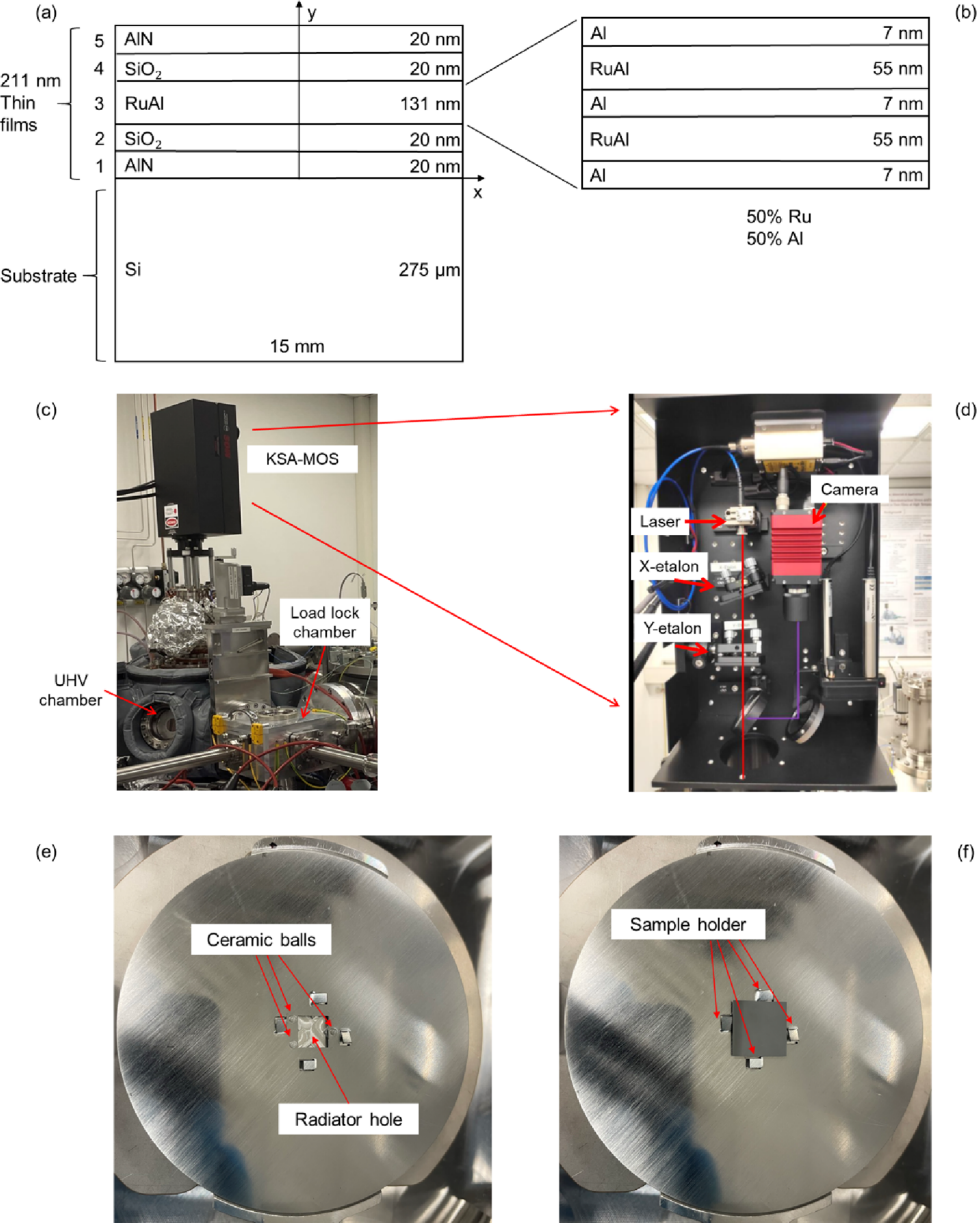
Multilayered thin film system analyzed in this study and instruments for thermal curvature measuring: (a) cross-section of sample; (b) material compositions of the RuAl alloy layer before alloying at elevated temperature; (c) KSA-MOS system connected on the UHV system; (d) internal structure of KSA-MOS system; (e) carrier in the UHV chamber without samples; (f) carrier in the UHV chamber with samples.

It was revealed in the previous studies that RuAl with two layers of AlN and SiO_2_ on its top and bottom as barrier and cover layers is a durable material for high temperature applications, including SAW sensors investigated at IFW Dresden.^20−24^ To implement the new model experimentally, a five-layered structure for the thin RuAl film shown in Figure 2a, on a square piece of Si substrate with lengths of 15 mm, is prepared.

The thin film shown in Figure 2a is composed of five layers from bottom to top (1 to 5). Layers 1 and 2 are barrier layers, which prevent the diffusion process between the substrate and the alloy, i.e., layer 3. Complementary, layers 4 and 5 are cover layers, which isolate the alloy layer 3 from air and prevent it from oxidation. The alloy layer 3 is the functional part. By deposition, it is composed of a stack of alternating RuAl and Al layers as shown in Figure 2b, which combine during the annealing process and form one layer of RuAl alloy.^15,21,22^

The Si substrate is cut to 15 mm square from a double-side polished Si wafer with the crystalline orientation of [100], so that Si shows isotropic thermo-mechanical behavior within the sample plane.^25^ The isotropy of film layers and substrate is important for the implementation of eq 19, which is modified under the equal-biaxial stress state of the multilayered system. With the structure designed above, the cut and cleaned Si substrates are coated with the thin films on the top surface by a magnetron sputtering deposition system.

### 3.2 Experimental Setups

In order to determine thermal stress in a multilayered thin film system, using eq 19, the sample curvature induced by temperature variations is required as an input parameter. In this study, an MOS system (K-Space associates, INC., USA) installed on a custom UHV chamber (CreaVac GmbH, Germany) shown in Figure 2c is used to measure the bending curvature of samples in a controlled environment in terms of pressure, temperature, and sample handling.

To stabilize the microstructure of as-deposited thin film layers, the samples were annealed at 600 °C for 10 h in the UHV chamber as suggested elsewhere.^20,23,24^ In the annealing process, temperature was increased with a rate of 6 K/min from 20 °C (room temperature) to 600 °C, then kept at 600 °C for 10 h, and then cooled down to room temperature. The whole annealing is carried out in UHV (10^–9^ mBar) to avoid reactions of the metals with air. After being annealed, the samples are ready for curvature measurement at varying temperatures.

The MOS system (Figure 2d) consists of a laser source, optical elements (etalons), and a CCD camera. In this system, the laser beam from the laser source is divided into multiple parallel beams by etalons and aimed at the surface of the sample in the UHV chamber (Figure 2f). The CCD camera monitors the spacing between the reflected beams from the sample. Due to the thermal stress, the sample bends, which reflects the beams with a different angle compared to the unbent sample. Thus, spaces between the reflected beam change and the curvature of the sample can be measured by the change in the spacing of the reflection spots.^26^

In order to measure the curvature caused by pure thermal stress, the sample has to bend freely during the temperature change, while the position of the sample has to be fixed to reflect the laser beams correctly. As shown in Figure 2e, the sample is supported by three ceramic balls. They only provide vertical reaction forces, so the sample can expand freely with as low a friction as possible on the smooth ceramic balls. To prevent the sample from slipping caused by vibrations, the sample holders (Figure 2f) are needed. They are slightly wider than those of the sample. Hence, the sample holders do not provide any reaction force to the sample. After putting the sample on the carrier and transfer to the measurement position, the chamber is vacuumed to 10^–9^ mbar, and the radiation heater under the radiator hole (Figure 2e) is started.

The temperature ramp provided by the heater is from 20 to 600 °C with 2 K/min rate. It is important to mention that the device can reach 900 °C in principle. However, to avoid delamination between films and the substrate, the maximum temperature is limited to 600 °C for our tested material systems. The curvatures of the samples are measured by the MOS system during the thermal process. In order to better implement and validate the analytical formula, the measuring points are picked at 20, 100, 200, 300, 400, 500, and 600 °C. After measurement, the thermal curvature is calculated by subtracting the curvature measured at 20 °C (reference point). The values of the acquired thermal curvature are shown in Supporting Information (Tables S1 to S3). For the reason for convenience, curvature 1/*r* appearing in later sections only stands for the thermal curvature.

### 3.3 Indirect Method for Temperature-Dependent Young’s Modulus Determination

In this section, a method to acquire Young’s modulus of the thin film at high temperatures (up to 600 °C in this study) based on curvature variations of the sample and by applying the multilayer-adapted Stoney equation is described.

In this method, which is called the curvature method, thermal curvature variations of a sample consisting of a thin film with unknown properties, deposited on a substrate with known properties, are measured. By changing the sample temperature, the multilayered sample will bend due to thermal stress, as discussed in previous sections. Thus, the curvature of the multilayered sample reflects the unknown thermo-mechanical properties of the thin film, if their relationship could be found.

In order to build the relation between the curvature radius and Young’s modulus of unknown thin film, eqs 11 and 19 are combined with considering equal-biaxial modulus as
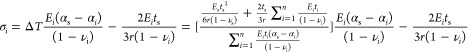
21

For a sample with a single film layer, eq 21 becomes
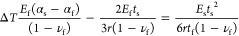
22Here, the film index is changed to f in the single-layer case. To express *E*_f_ (Young’s modulus of the unknown film), eq 22 is rewritten as
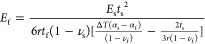
23

To implement eq 23, the unknown parameters of the film material on the right-hand side of eq 23 are to be acquired before deposition on the substrate. From a practical point of view, the Poisson ratio ν_f_ is assumed constant during the thermal process. Hence, the Poisson ratio of the unknown film is obtained at room temperature, which can be acquired for example by a tensile test. The CTE of film α_f_ can also be measured by a CCD camera at different temperatures.^27,28^ With the required parameters, eq 23 gives the relation between unknown Young’s modulus (*E*_f_) and measured sample curvature (1/*r*) in different temperatures as
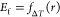
24

Using eq 23, Young’s modulus of any thin film deposited on a known substrate can be calculated by measuring the variation of sample curvature due to thermal stress. In addition, eq 23 also provides a functional relationship varying with Δ*T*, which determines Young’s modulus (*E*_f_) as a function of temperature. The validation of the curvature method is shown in the results section.

### 3.4 Numerical Simulation

A 3-D finite element (FE) simulation of the multilayered thin film sample was developed in COMSOL 5.6 software. In this model, all of the specifications including the sample geometry, boundary conditions (BCs), loads, etc. were set as close as possible similar to the experiment described in section 3.2.

**Figure 3:**
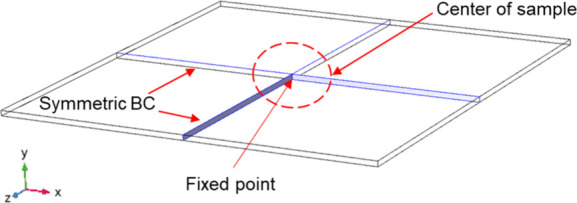
Boundary conditions of FE simulation of the thin film system.

To simulate the free-expand condition of the sample, we have implemented symmetric boundary conditions with a fixed point to eliminate rigid-body motions, which serve a function similar to that of sample holders in our experiments. Figure 3 shows the boundary conditions applied to the model. Because of the symmetric geometry of our samples, the symmetric BCs with a fixed origin point are applied on the cross sections of the *x*–*y* and *y*–*z* planes, which limit the rotations and displacements at the origin point.

The temperature-dependent material parameters are imported for each film layer and the substrate from the literature and the Material Properties Database (MPDB) (the material properties are presented in S4).^29−33^ Additionally, for the RuAl alloy layer (Figure 2b), Young’s modulus determined by the curvature method (shown in the results section) is applied to the model.

Linear cubic elements are applied to structured grids with the number 31370. A mesh convergence study was also performed to eliminate the effect of elemental shapes and volume on the simulation results. Based on our quasi-static experimental processes, the linear stationary analysis with homogeneously distributed thermal load input from 20 to 600 °C is chosen for the FE solver. The stress at the center of the sample is investigated, where stress distributes linearly due to symmetric BCs. The FE model is validated by comparing the thermal curvature obtained from the simulation and the experiments, which is explained in section 4.2.

## 4 Results and Discussion

With the curvature data (shown in the SI) acquired by the custom measurement setup, the thermal stress in each layer of the five-layered sample (Figure 2a) is calculated by the derived equation in this study for the multilayered thin film systems (i.e., eq 19). The results are shown in Table 1, in which the thermal stress means the representative film stress σ_*i*_ in layer “I”, and the negative stress shows the compressive state of the stress.

**Table 1:** Thermal Stress of the Five-Layered RuAl Sample during Thermal Variations from the Multilayer-Adapted Stoney Equation

	thermal stress (MPa)				
temperature (°C)	AlN Layer 1	SiO_2_ Layer 2	RuAl Layer 3	SiO_2_ Layer 4	AlN Layer 5
20^a^	0	0	0	0	0
100	–23.23	16.39	–142.20	16.39	–23.23
200	–51.79	39.57	–368.25	39.57	–51.79
300	–85.27	65.42	–557.32	65.42	–85.27
400	–127.04	94.70	–718.41	94.70	–127.04
500	–166.75	119.61	–846.92	119.61	–166.75
600	–217.49	149.71	–994.95	149.71	–217.49

a Ambient temperature.

From Table 1, it can be noticed that the values of thermal stress are the same in layers 1 and 5 and in layers 2 and 4, which consisted of the same materials. This means that the thermal stress within the film layer with the same material is identical. This could be concluded that in a multilayer thin film system with a high thickness ratio of the substrate to the total film layer (e.g., in this study the total film thickness is 211 nm and the substrate thickness is 275 μm), which is bent due to the thermal stress, the stress value within different film layers only varies with the film material properties.

### 4.1 Results of the Curvature Method for Young’s Modulus Determination

To show the accuracy of the proposed method for the calculation of Young’s modulus of thin films at temperatures higher than room temperature (called the curvature method), Young’s modulus of an already known material was obtained by the curvature method and compared with the reported values in the literature.

**Figure 4:**
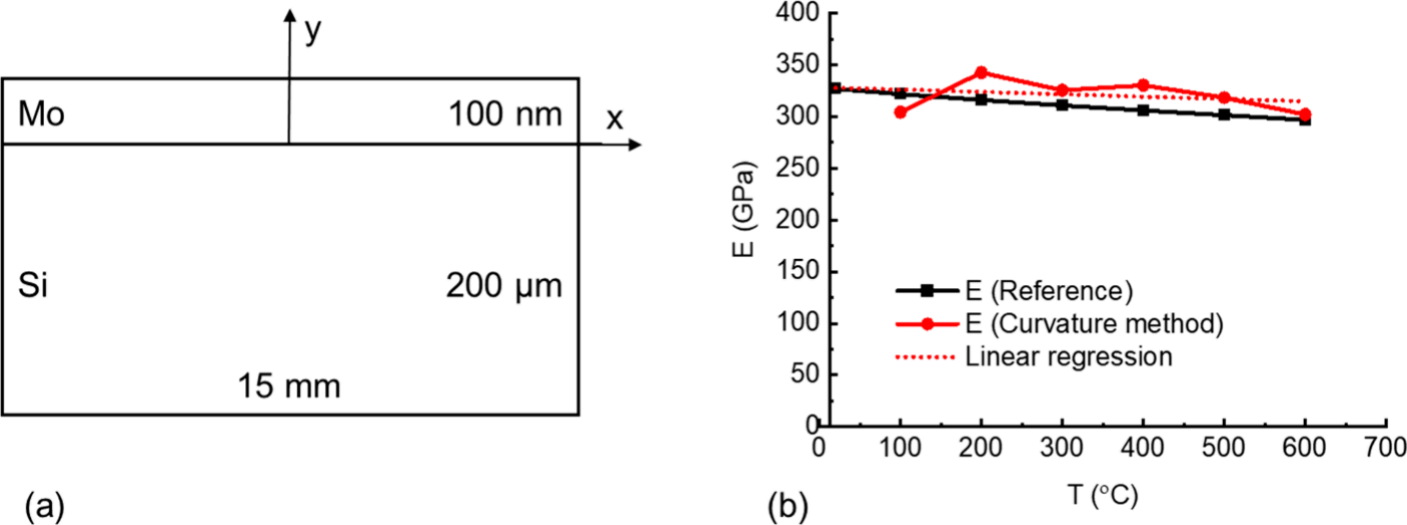
Validation of the curvature method: (a) reference sample; (b) Young’s modulus–temperature curve of Mo thin film.

The curvature values measured from the experiment on a single-layer thin film sample of a well-known material (molybdenum (Mo)) on the Si substrate, which is called a “reference sample”, were used to calculate Young’s modulus of the thin film at different temperatures by eq 23. To prepare the reference sample, a single Mo layer with a thickness of 100 nm was deposited on a square Si substrate with side lengths of 15 mm, as shown in Figure 4a. Then, the curvature variations of the reference sample during the same temperature variations that applied to the RuAl sample (i.e., between 20 and 600 °C) were measured by the MOS system and shown in the SI. The calculated values of Young’s modulus using eq 23 (labeled as “E (curvature method)”) at different temperatures and the corresponding values obtained from literature^18,34^ (labeled as “E (Reference)”) are presented in Figure 4b and Table 2.

**Table 2:** Young’s Modulus of Mo and RuAl Thin Film

	Mo thin film			RuAl thin film
temperature (°C)	E (Reference) (GPa)	E (curvature method) (GPa)	relative error	E (curvature method) (GPa)
20	326.91	undefined	undefined	undefined
100	321.79	304.31	5.43%	540.72
200	316.02	342.58	8.40%	666.53
300	310.80	325.53	4.74%	654.86
400	305.96	330.34	7.97%	600.30
500	301.35	318.36	5.64%	563.94
600	296.81	302.01	1.75%	526.46

It can be noticed that Young’s modulus calculated by the curvature method at 20 °C is undefined. This is because this temperature is the reference temperature, where Δ*T* and 1/*r* are zero. Therefore, the calculation near the reference temperature should be avoided in the curvature method. The relative errors between E (reference) and E (curvature method) shown in Table 2 are smaller than 10%, which indicate the accuracy of the proposed curvature method for the calculation of Young’s modulus of thin film at high temperatures.

A curve was fitted using the linear regression method to the E (curvature method) values, which were calculated for the Mo thin film, as shown in Figure 4b. The equation for the fitted curve is
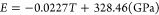
25where *T* is the temperature in °C. Then, Young’s modulus of Mo thin film at room temperature (i.e., 20 °C) was estimated using eq 25. The estimated value is 328.01 GPa, which is close to the corresponding value obtained from the literature, which is 326.91 GPa, with a relative error equal to 0.34%. This linear regression covers the shortage of the curvature method, so that it can be applied at the reference temperature.

The investigation of the curvature method for Mo thin film as a material with known properties at high temperatures shows that the method is trustable for the acquisition of Young’s modulus of thin films, for which no data are available in the literature. Therefore, we acquired Young’s modulus of RuAl thin film at temperatures higher than room temperature, and the values are presented in the last column of Table 2. These values are then used as a material input for the FE simulation of the thermal stress distribution in the multilayered thin film.

### 4.2 Comparison of Analytical and Numerical Methods

**Figure 5:**
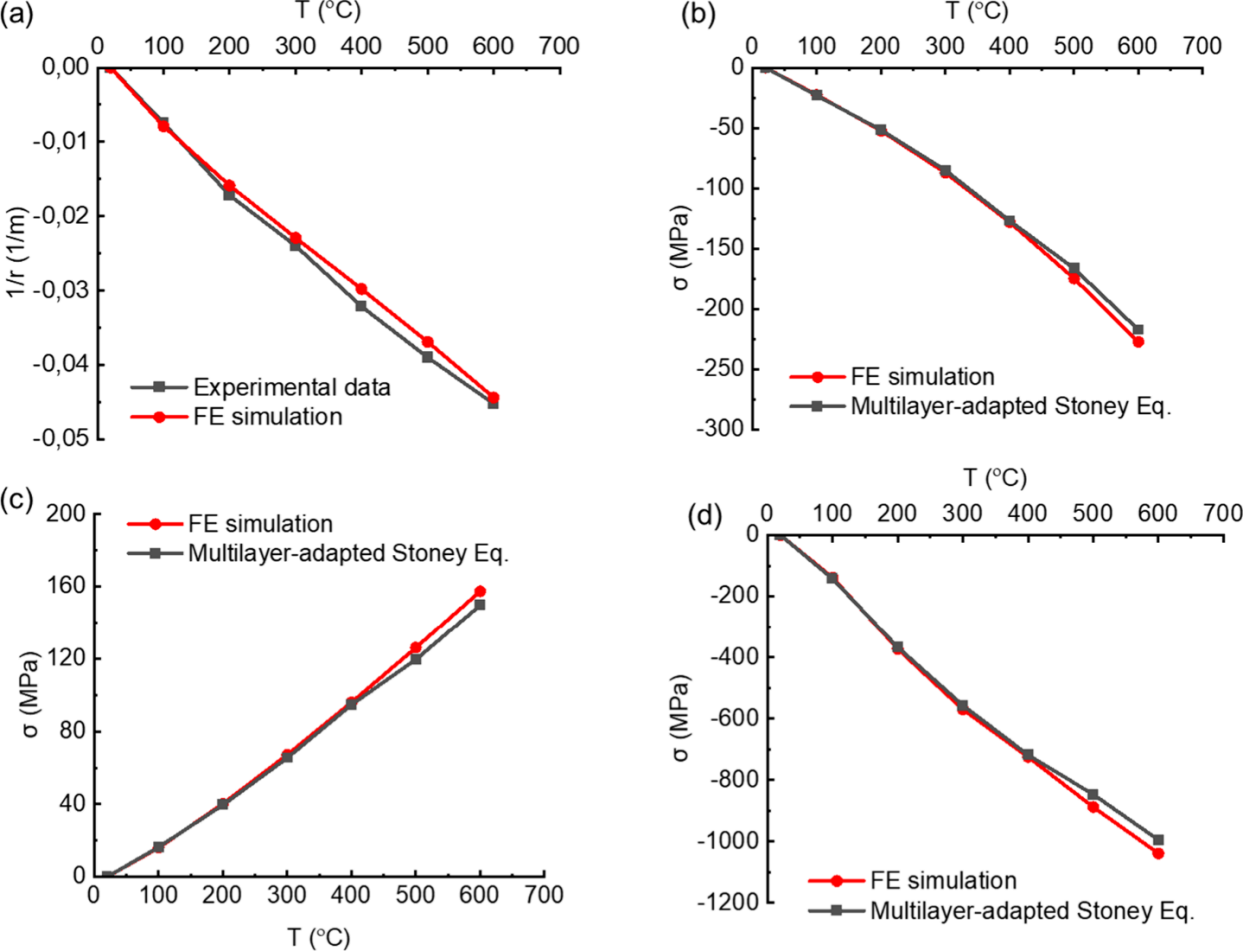
Validation of the FE model and thermal stress comparison between FE simulation and multilayer-adapted Stoney equation for thin films in a RuAl-based multilayered sample at different temperatures: (a) curvature–temperature curve of reference sample; (b) film stress in AlN Layer 1; (c) film stress in SiO_2_ Layer 2; (d) film stress in RuAl Layer 3.

The results of thermal stress in the multilayered thin film system obtained from the multilayer-adapted Stoney equation (eq 19) and the FE simulation are compared to investigate the accuracy of both methods. Therefore, first the FE model is validated by comparing it with the experimental curvature data obtained from the reference sample of a single-layered Mo film on the Si substrate, which was explained in the previous section. During the temperature variations from 20 to 600 °C, the curvature variations of the reference sample were measured by the custom MOS system and compared with the curvatures obtained from the FE simulation. As shown in Figure 5a, the FE simulation results are close to the experimental results, which means that the FE model is validated.

The thermal stress values in the bottom of each film layer of the five-layered system are extracted from the validated FE simulation and compared with the results calculated by the multilayer-adapted Stoney equation shown in Table 1. In the five-layered system shown in Figure 2a, there are only three different film materials because we observed that the stress in the thin films with the same materials in the layered system is identical; therefore, it is sufficient to compare the thermal stress obtained from the analytical and the numerical methods in each of layers 1, 2, and 3 as shown in Figure 5b–d.

Figure 5b–d shows that the thermal stresses that were calculated by the multilayer-adapted Stoney equation (eq 19) match with the stresses obtained from the FE simulation.

## 5 Conclusions

In this paper, the thermo-mechanical properties of multilayered thin film stacks on a substrate are investigated. To obtain the thermal stress in each film layer, the multilayer-adapted Stoney equation is derived and validated by FE simulation. To obtain the temperature-dependent Young’s modulus of unknown film materials, the curvature method is developed and validated by a reference sample.

Compared with the conventional thermal stress formula derived by Hsueh (eq 20), the multilayer-adapted Stoney equation allows the calculation of the thermal stress in a multilayered thin film system with the sample curvature from a single experiment. This advantage reduces the number of experiments from *n* + 1 to 1 for thermal stress calculation in an *n*-layered thin film system, which not only saves the multiple experimental time and costs but also makes the real-time thermal stress analysis with the in situ sample possible.

If the film material is unknown, the new model can be restructured to develop the curvature method, which provides a new way to measure Young’s modulus by measuring the sample curvature. This method can be used to acquire temperature-dependent Young’s modulus, where a conventional method like the tensile test or nanoindentation is not suitable.

There are also limitations. Since the thermal stress calculated here is a first-order approximation, still some amounts of errors exist. However, in our implementations, the film is thin enough, and the errors can be neglected. However, when the film thickness is close to the substrate thickness, the multilayer-adapted Stoney equation is no longer suitable. In order to limit the parameters of the model for engineering implementations, the influence of layer interfaces and also the effect of the film–substrate interface, such as roughness, mismatches, delamination, sliding, etc., are not considered. This assumption agrees with the displacement continuity assumption that has been considered in previous research on the investigation of stress in thin films.^9−14^ The influence of interfaces should be examined in a future study. In addition, morphology changes in the tested material systems should be avoided during temperature changes. For the curvature method, there is also a drawback. When the temperature is close to the reference temperature, Young’s modulus cannot be measured by the curvature method. The inconsistency in the curvature method between 20 and 100 °C resembles the limits of the measurement accuracy of the setup and is attributed to the small value of the thermally induced sample curvature in combination with the limits of the heating system used. In order to increase the feasible range of the original curvature method, additional approaches, such as regression analysis, need to be implemented. For the material investigated in this study, a simple linear regression is acceptable.

For future research, the multilayer-adapted Stoney equation can be used as a valid method to acquire thermal stress within a multilayered thin film system. The curvature method also provides a valid indirect method to acquire the temperature-dependent Young’s modulus of an unknown material. Additionally, the FE model presented can be used to study the stress distribution in a multilayered system with complex geometry and anisotropic substrate, for example, the thermal stress within comb-shaped structured IDTs of SAW devices.

## Supporting Information Available

(1) Curvature for thermal stress calculation; (2) data for validation of curvature method and FE model; (3) curvature for determination of temperature-dependent Young’s modulus of RuAl; (4) material properties (PDF)
Temperature-dependent material parameters of AlN, SiO_2_, RuAl, silicon [100], and molybdenum varying with temperature *T* in (K) (ZIP)
