# Supplementary material for: A Practical Approach for Determination of Thermal Stress and Temperature-Dependent Material Properties in Multilayered Thin Films
Source: ACS Appl Mater Interfaces. 2024 Jun 10;16(24):31729–37. doi: 10.1021/acsami.4c03166 (PMC11194766; doi:10.1021/acsami.4c03166)
Supplement: Supplementary file 1 — am4c03166_si_001.pdf [file am4c03166_si_001.pdf]

Supporting Information

A Practical Approach for Determination of Thermal Stress and Temperature-Dependent Material Properties in Multilayered Thin Films

Yanqiao Yang, Andreas Winkler, Atefeh Karimzadeh*

Leibniz IFW Dresden, SAWLab Saxony, Institute for Emerging Electronic Technologies (IET), Group "Acoustic Microsystems", Helmholtzstr. 20, 01069 Dresden, Germany.

*Corresponding author: a.karimzadeharani@ifw-dresden.de

Table

Table S1. Curvature of five-layered RuAl sample during thermal variations measured by custom MOS system for the thermal stresses calculation.

Temperature (℃)	Total curvature (1/m)	Thermal curvature (1/m)
20	0.0326	0
100	0.0242	-0.0084
200	0.0109	-0.0217
300	-0.0006	-0.0332
400	-0.0107	-0.0433
500	-0.0191	-0.0517
600	-0.0290	-0.0616

Table S2. Curvature of reference sample with single Mo layer during thermal variations measured by custom MOS system for the validation of the curvature method and FE model.

Temperature (℃)	Total curvature (1/m)	Thermal curvature (1/m)
20	0.0549	0
100	0.0474	-0.0075
200	0.0377	-0.0172
300	0.0309	-0.0240
400	0.0227	-0.0322
500	0.0159	-0.0390

600	0.0096	-0.0452

Table S3. Curvature of sample with single RuAl layer during thermal variations measured by custom MOS system for the determination of temperature-dependent Young's modulus of RuAl.

Temperature (℃)	Total curvature (1/m)	Thermal curvature (1/m)
20	0.0561	0
100	0.0480	-0.0081
200	0.0341	-0.0218
300	0.0224	-0.0337
400	0.0129	-0.0432
500	0.0026	-0.0535
600	-0.0073	-0.0634

Material

Material S4.zip. Temperature-dependent material parameters of AlN, SiO2, RuAl, Silicon [100] and Molybdenum variating with temperature T in (K).
